# A Canine Non-Weight-Bearing Model with Radial Neurectomy for Rotator Cuff Repair

**DOI:** 10.1371/journal.pone.0130576

**Published:** 2015-06-24

**Authors:** Xiaoxi Ji, Nirong Bao, Kai-Nan An, Peter C. Amadio, Scott P. Steinmann, Chunfeng Zhao

**Affiliations:** 1 Biomechanics Laboratory, Division of Orthopedic Research, Mayo Clinic, Rochester, MN, 55905, United States of America; 2 Trauma Center, Shanghai First People’s Hospital, Shanghai Jiao Tong University, Shanghai, 200080, China; University of Insubria, ITALY

## Abstract

**Background:**

The major concern of using a large animal model to study rotator cuff repair is the high rate of repair retears. The purpose of this study was to test a non-weight-bearing (NWB) canine model for rotator cuff repair research.

**Methods:**

First, in the in vitro study, 18 shoulders were randomized to 3 groups. 1) Full-width transections repaired with modified Mason-Allen sutures using 3-0 polyglactin suture, 2) Group 1 repaired using number 2 (#2) polyester braid and long-chain polyethylene suture, and 3) Partial-width transections leaving the superior 2 mm infraspinatus tendon intact without repair. In the in vivo study of 6 dogs, the infraspinatus tendon was partially transected as the same as the in vitro group 3. A radial neurectomy was performed to prevent weight bearing. The operated limb was slung in a custom-made jacket for 6 weeks.

**Results:**

In the in vitro study, mean ultimate tensile load and stiffness in Group 2 were significantly higher than Group 1 and 3 (p<0.05). In the in vivo study, gross inspection and histology showed that the preserved superior 2-mm portion of the infraspinatus tendon remained intact with normal structure.

**Conclusions:**

Based on the biomechanical and histological findings, this canine NWB model may be an appropriate and useful model for studies of rotator cuff repair.

## Introduction

Rotator cuff tears are a common musculoskeletal disorder, affecting 9.4% to 39.0% of the general population, with increasing occurrence among older individuals [1–4]. Surgical repair of a torn rotator cuff is considered the criterion standard for relieving pain and restoring shoulder function, but the retear rate ranges from 17% to 94%[5–7]. A number of mechanical and biological strategies have been developed to prevent retears, including improved suture techniques, bone substitutes, periosteum autografts, growth factors, gene therapy, stem cell transplantation, and others[8–11], but a critical need remains for appropriate animal models to investigate the efficacy of a specific improvement approach or the tendon-to-bone healing process.

To mimic the features of human injuries, the ideal animal model must possess similar soft tissue and bony anatomy, a chronic injury condition, a proper tendon size for standard-of-care repair techniques, and a healing ability similar to humans[12,13]. Current animal models include the rat, rabbit, goat, and sheep, but the canine model appears advantageous in studies of various augmentations and grafts, different repair techniques, and novel stimulation treatments[14–21]. The relatively large size of the rotator cuff facilitates accurate and reproducible acute injuries and repair manipulations[22], and postoperative rehabilitation methods, including casting, slinging, and treadmill running, are tolerated[23–25].

However, the shortcomings of the canine model cannot be ignored, particularly the high retear rate after rotator cuff repair. Some have reported that acute, full-width tendon repairs failed universally within the first postoperative days, regardless of suture type, suture configuration, or postoperative protocol[12]. Robust scar tissue fills the gap between the tendon end of the failed repair and the humerus. This finding is supported by other studies both in the dog and in sheep models, which show scar tissue filling the gap between tendon and bony tissue[16,26–28]. The canine model still needs further improvement and modification if it is to be used to simulate human rotator cuff injury and repair.

A canine model with a high level of radial nerve denervation has been well developed for flexor tendon research[29,30]. This model effectively prevents weight bearing and allows execution of a postoperative rehabilitation regimen. Although this non–weight-bearing (NWB) canine model has been successfully used for flexor tendon research, it has not been tested in a rotator cuff repair model. The purpose of this study was to test the feasibility of a canine NWB model for studies of rotator cuff repair. Since one of our current ongoing studies was using NWB model for another purpose of the research, it would be possible to test this model for a rotator cuff injury. However, it was not acceptable for doing a major surgery (rotator full laceration and repair) as a side project in the same dog from animal care perspective although it did confound the main project. But a simple procedure, such as a partial tendon cut without repair, would be acceptable by the Institutional Animal Care and Use Committee. Therefore, in the current study, we designed a study to use a partial tendon laceration alone to test if the NWB canine model would be appropriate for rotator cuff repair. Our working hypothesis was if the failure (rupture) strength of a tendon partial laceration would be equivalent to or less than a full-laceration with surgical repair and if this partial laceration model would be sustained intact without rupture in a NWB model in vivo, this NWB could be safely used for rotator cuff repair either in partial or full transection model. To test this hypothesis, first a full-width transected infraspinatus (IS) tendon repaired with different sizes of suture and a partial-width transected tendon without repair were tested in failure strength using in vitro model. Then, the partial transected tendon without repair was tested in a NWB model in vivo after 6-week postoperative observation.

## Material and Methods

This study was approved by the Mayo Clinic Institutional Animal Care and Use Committee (IACUC). All vendors were listed as Class A Dealers by USDA.

### In Vitro Study

#### Creating and Repairing Rotator Cuff Tears

**Fig 1:**
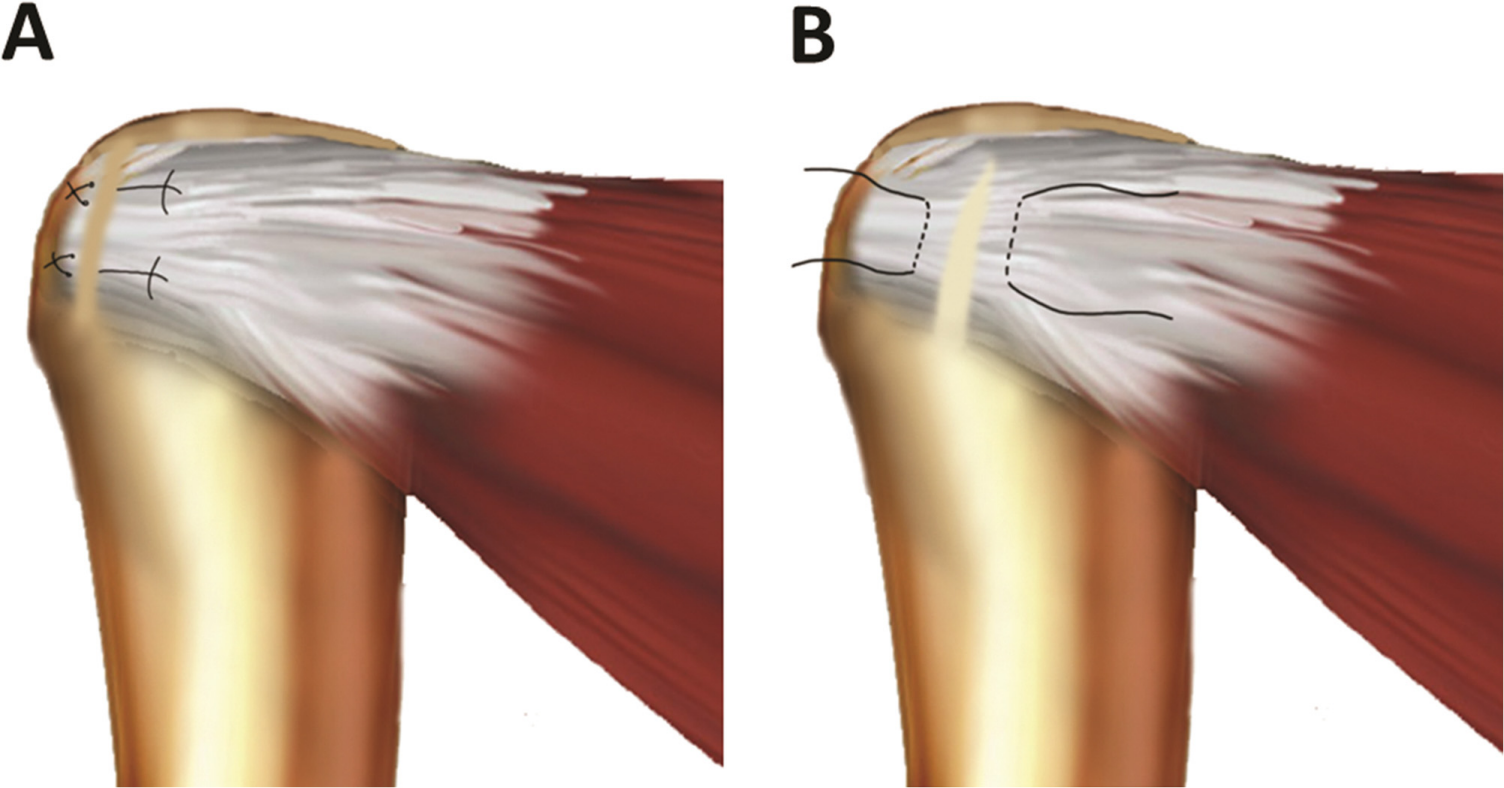
A, Full-width transection of the infraspinatus (IS) tendon and repair with modified Mason Allen sutures (3–0 polyglactin or #2 polyester braid and long-chain polyethylene) in the in vitro study. B, Partial-width transection of IS tendon and placement of 2 marker sutures in the in vivo study.

Eighteen shoulders specimens were harvested from mixed-breed dogs (average weight, 20 kg) that were euthanized for other studies approved by our Institutional Animal Care and Use Committee. All muscle attachments, except for the IS tendon, were detached from the humerus for each shoulder. Following a previously described model [31], a full-width transection was created by first identifying the anterior and posterior extent of the IS tendon and then sharply detaching the tendon from the bone surface in its entirety. Partial transections were created by sharply releasing the IS tendon from the inferior edge while preserving the superior 2 mm of the tendon (Fig 1). Our pilot trials indicated that this partial transection had mechanical strength that was similar to or less than that of an IS tendon after full-width transection and repair.

Modified Mason-Allen sutures were used to repair the IS tendon to the greater tuberosity. We used 3–0 polyglactin sutures (Vicryl; Ethicon, Bridgewater, NJ) or polyester braid and long-chain polyethylene sutures (Fiberwire #2; Arthrex, Inc., Naples, FL)[32–35] to compare the strength of rotator cuff repair with different sizes of suture. The 18 shoulders were randomly assigned to 3 groups (6 specimens per group): 1) full transection of the IS tendon and repair with 3–0 sutures; 2) full transection of the IS tendon and repair with #2 sutures; and 3) partial transection of the IS tendon without repair (preserving the superior 2 mm width of the tendon).

#### Mechanical Testing

After creating and repairing rotator cuff tears, specimens were mounted on a servohydraulic testing machine (858 MiniBionix II, MTS Systems Corp., Eden Prairie, MN) for mechanical evaluation. A custom-made clamp gripped the IS tendon. The humerus was potted into a small block of polymethylmethacrylate, mounted, and positioned at an incline of 135° to the long axis of the tendon to model the physiologic pull of the IS tendon. Each repaired rotator cuff specimen was loaded to failure under displacement control at a rate of 0.5 mm/sec. Load and actuator displacement were recorded at a sample rate of 50 Hz. Ultimate tensile load was defined as the peak force observed during loading. Each specimen’s failure mechanism (tendon breakage, suture breakage, suture pullout through tendon or bone tunnel breakage) was also recorded.

#### Statistical Analysis

In addition to reporting means and standard deviations, 1-way analysis of variance was used to analyze differences in ultimate tensile load and stiffness (JMP software; SAS Institute Inc., Cary, NC). If a significant difference was detected, the Studentized range, Tukey honestly significant difference post hoc test was used to assess the biomechanical properties of each group. Statistical significance was set at *P* < .05 in all cases.

### In Vivo Study

#### Surgical Procedures

Six mixed-breed dogs (average weight, 20 kg) obtained from the venders that have been licensed by Office of Laboratory Animal Welfare (OLAW) and United States Department of Agriculture (USDA) were used for this portion of the study. Morphine 0.5 mg/kg was administrated intravenously 30 minutes preoperatively. Dogs were anesthetized using intravenous ketamine (10mg/kg) and diazepam (0.6mg/kg) plus isoflurane inhalation during the surgical procedure. The IS tendon was exposed through a 5-cm transverse incision at the acromion. The IS tendon was sharply transected from the inferior edge, with the superior 2 mm of the tendon intact near the insertion. Two 2–0 polyglactin sutures (Vicryl; Ethicon, Inc., Bridgewater, NJ) were placed as markers at the 2 transected tendon ends to identify the laceration 6 weeks later (Fig 1).

After partial transection of the IS tendon, the radial nerve proximal to the triceps innervation was exposed through a lateral humeral incision. Neurectomy was performed to denervate the elbow and wrist extensors to prevent postoperative weight-bearing and potential muscle contraction on the operative limb. This protocol has been well established in the canine model of flexor tendon repair[36,37]. Although neurectomy effectively limits elbow and wrist active extension, animals can drag their limb on the ground which may cause limb or forepaw injury. Furthermore, some animals are able to passively extend the joint, which may cause repaired tendon rupture. Therefore, the operated limb was loosely slung in a custom denim jacket to further protect the operated limb from injures during cage activities.

#### Postoperative Procedure

After surgery, the dogs were kept in a recovery room for 24 hours of intensive care and then allowed free activity in a large cage. Buprenorphine (slow release formula) 0.01 mg/kg was given subcutaneously once postoperatively, and then Carprofen 4 mg/kg subcutaneously daily for 1 week postoperatively. Rehabilitation began on postoperative day 3 for each dog. The operated shoulder was passively moved through flexion and extension, 20 repetitions daily, 7 days per week. Dogs were allowed free field (hallway) activity without the jacket for about 30 minutes daily. During this active motion period, dogs were closely observed to ensure the absence of weight bearing on the operated limb. The jacket would be placed back on the dog if any attempt of weight-bearing on the operated limb was noticed.

#### Gross Observation and Histologic Evaluation

All 6 dogs were euthanized with overdose of Pentobarbital through intravenous injection 6 weeks after surgery. The IS tendons of the operated and contralateral shoulders were carefully dissected from surrounding tissues for gross observation. The IS tendons then were transected and fixed in formalin, embedded in paraffin and cut into 7-μm sections. Hematoxylin and eosin staining was used to evaluate cell morphology and distribution of tendon and scar tissues.

## Results

### In Vitro Study

The mechanism of failure for all specimens in the 3–0 and #2 repair groups was suture pullout through the tendon, without suture or bone tunnel breakage. For specimens in the partial-transection group, failure occurred through rupture of the 2-mm preserved portion of the tendon.

**Fig 2:**
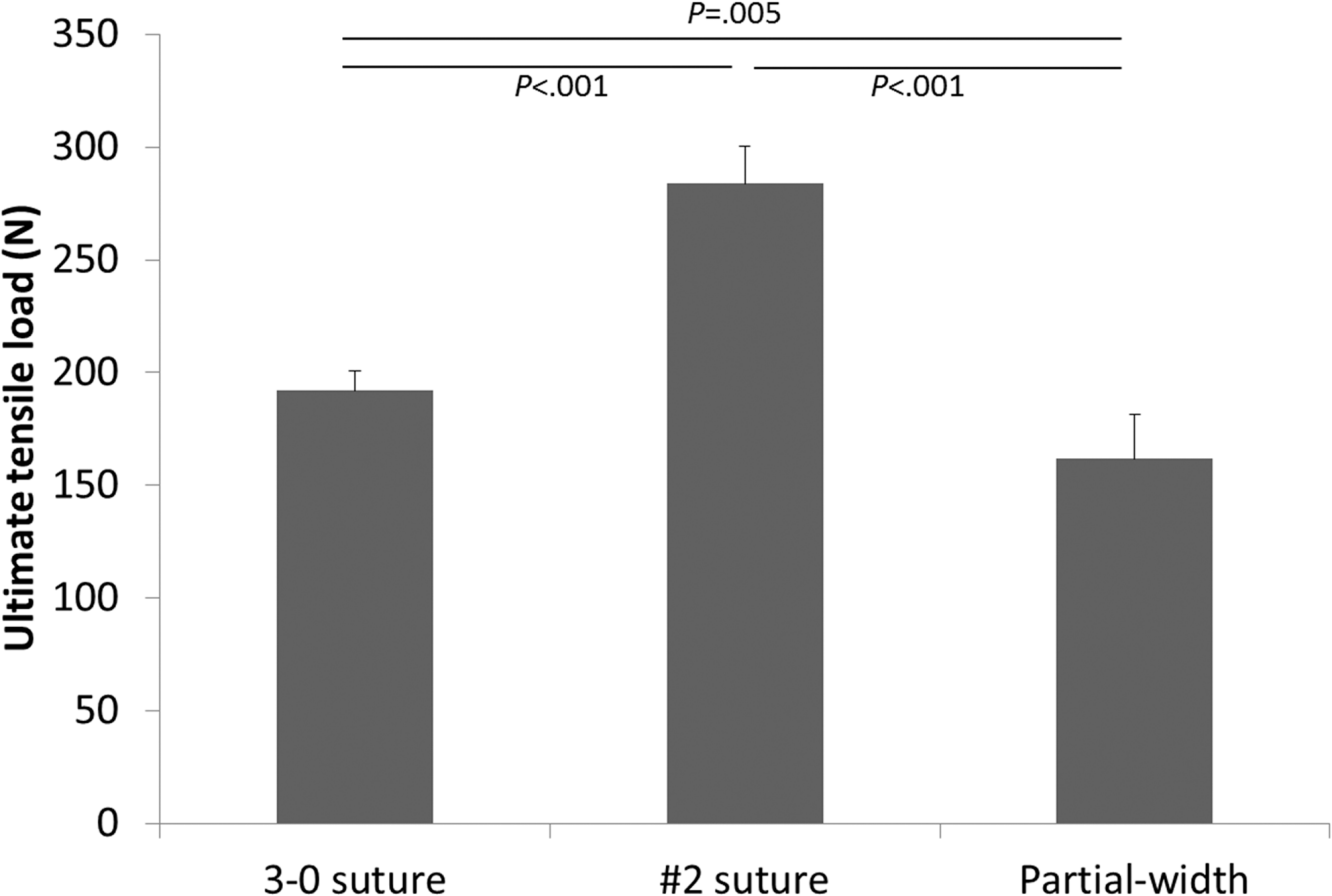
Comparison of ultimate tensile load of the 3–0 repair group, #2 repair group, and partial-width transection group.

**Fig 3:**
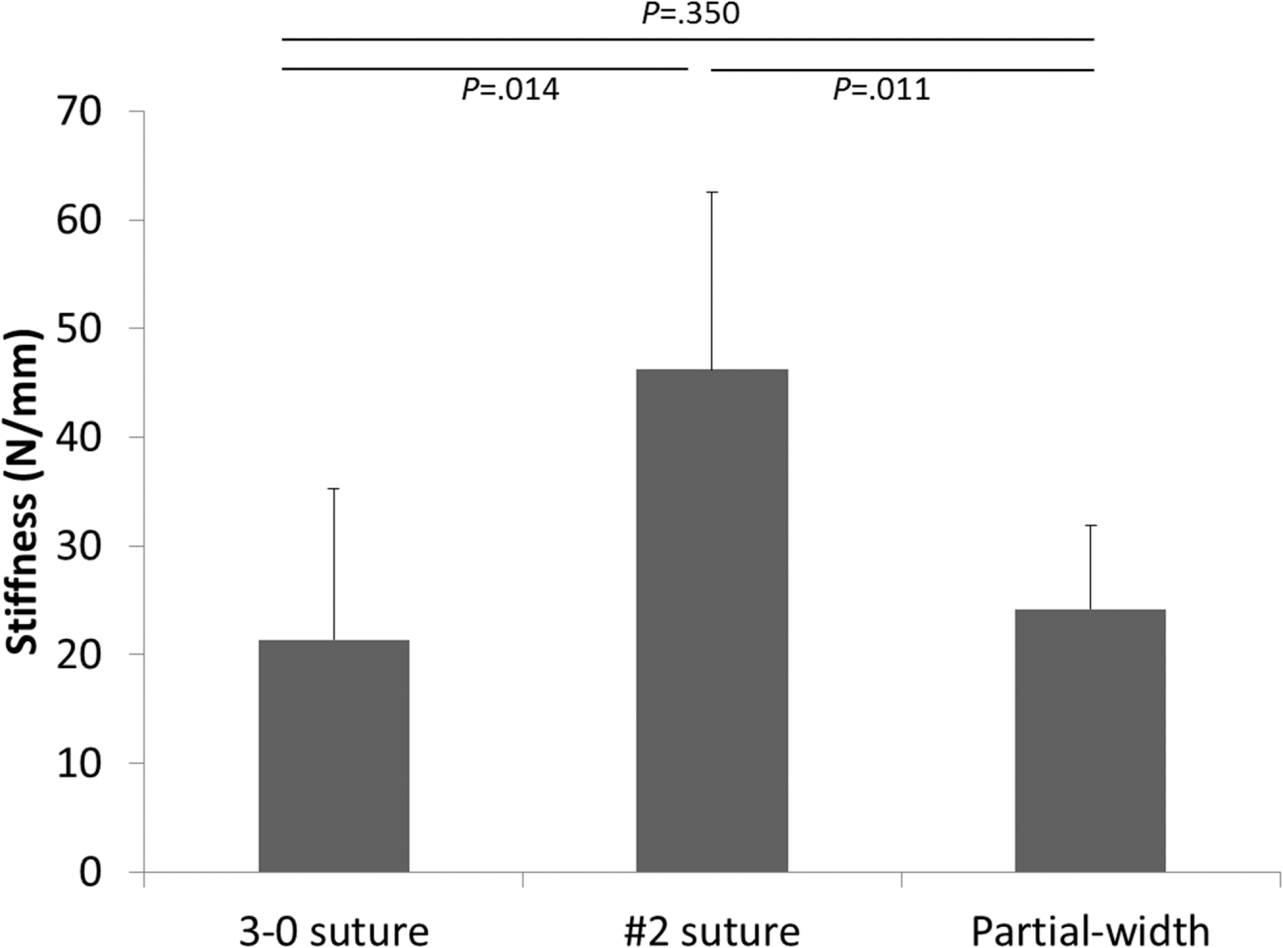
Comparison of stiffness of the 3–0 repair group, #2 repair group, and partial-width transection group.

Ultimate tensile load in the #2 repair group (mean [SD], 283.81 [16.50] N) was significantly higher than that of the 3–0 repair group (192.05 [8.42] N; *P* < .001) and the partial-width transection group (161.76 [19.56] N; *P* < .001) (Fig 2); the difference between the 3–0 repair group and the partial-width transection group also was significant (*P* = .005). Stiffness of the #2 repair group (mean [SD], 46.21 [16.34] N/mm) was significantly higher than that of the 3–0 repair group (21.34 [13.95] N/mm; *P* = .014) and the partial-width transection group (24.17 [7.71] N/mm; *P* = .011) (Fig 3). Stiffness measurements of the 3–0 repair group and the partial-width transection group were not significantly different (*P* = .350).

### In Vivo Study

**Fig 4:**
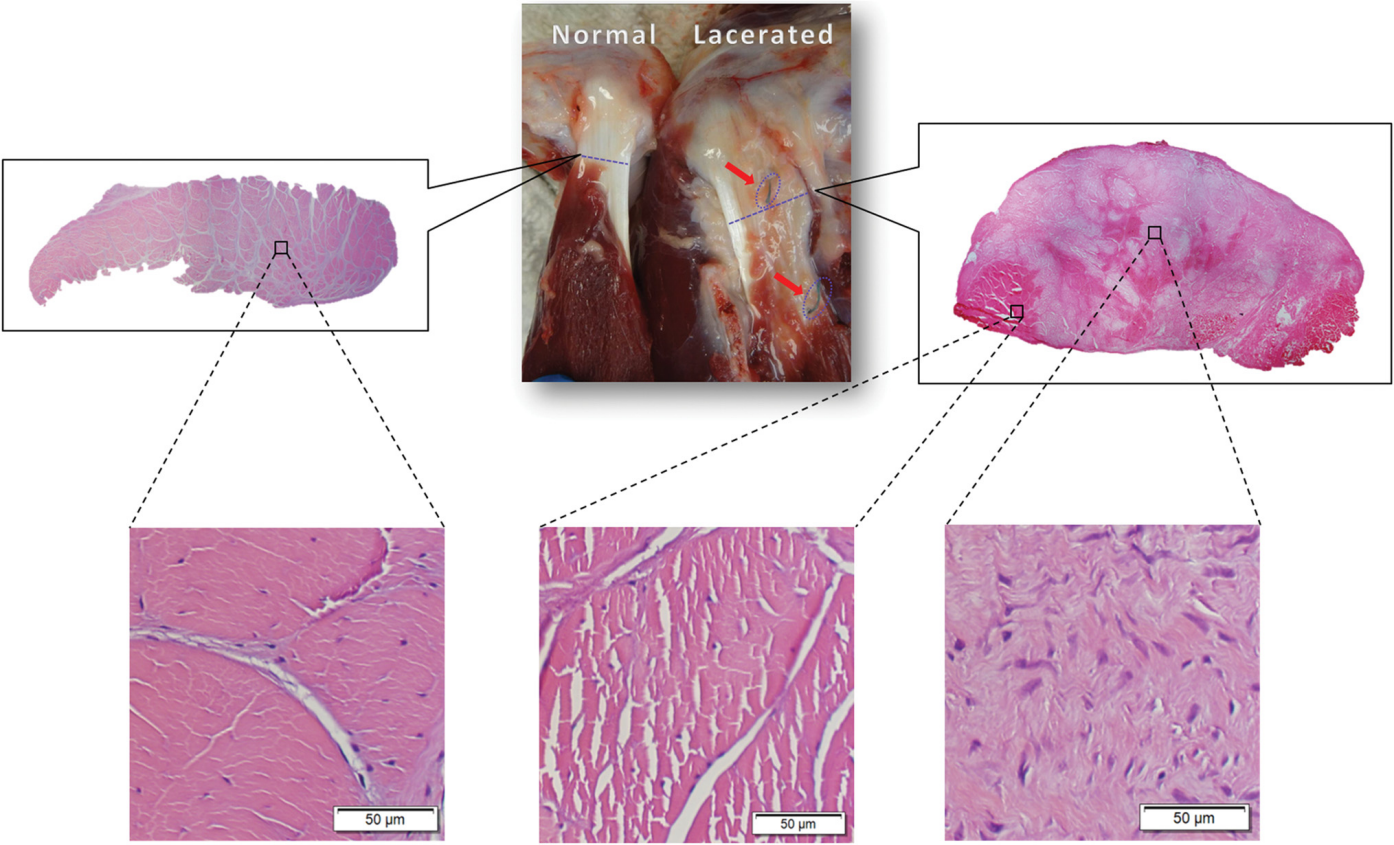
Gross inspection showed intact tendons 6 weeks postoperatively. Hematoxylin and eosin staining showed the scar tissue surrounding the normal tendon tissue with obvious margin. Red arrows indicate location of marker sutures.

Gross inspection showed that the preserved 2-mm superior portion of IS tendon was intact in all operated shoulders. However, the transected portion had retracted to form a 1- to 2-cm gap, as shown by the increased distance between the sutures placed at the surgical margins. Abundant scar tissue filled this gap without any discontinuity in the initial tendon transection. Excessive scar tissue seemed to increase the size of the construct and made it difficult to isolate the healed area from the normal tendon tissue. However, a clear margin between scar tissue and normal tendon was easily identified (Fig 4). Hematoxylin and eosin staining showed that the majority of tissue at the transection site was scar tissue, which surrounded the normal tendon tissue with an obvious margin (Fig 4).

## Discussion

The main limitation of a canine model for rotator cuff injury is the quadrupedal gait, which bears weight on both forelimbs and hind limbs. Bearing weight on an operated limb might put considerable tension on the repaired rotator cuff, resulting in a high retear rate[12,38]. Similar observations (high postoperative retear rate) also were reported for the limited-activity sheep model[26–28]. Some studies attempted to achieve better healing and less retear by reducing the load on repairs, by using bandages or slings to prevent the operated limb from bearing weight[12,17,18]. However, the outcomes of these procedures were not satisfied because a portion of repairs failed after only mild contracture of the shoulder joint. Although movement of the operated limb was limited by bandages or slings, the possibility of muscle and joint contraction and partial weight bearing still existed. Furthermore, a canine model of partial (two-thirds) release of the IS tendon with limited activity showed a 100% retear rate[16], suggesting that weight-bearing might have a very important role in rotator cuff retear.

The NWB model using radial neurectomy has been used successfully for years in studies of canine flexor tendon repair[36,37,39]. This method is well tolerated, safe, and reliable and avoids use of a postoperative spica cast. As the level of the innervation of the radial nerve is lower than the shoulder, the effects of this denervation on rotator cuff healing are minimal. This model reliably avoids weight-bearing on the limb and allows performance of postoperative rehabilitation, thus avoiding joint contracture and adhesion formation in the operated shoulder.

The current study showed the preserved 2 mm partial-width of IS tendon remained intact after 6 weeks in the current NWB canine model, which indicated that force applied to the IS tendon in this in vivo model was below the threshold of breaking strength of this partial preserved IS tendon. This partial tendon breaking strength (161 Newton) was lower than the both repairs in a full transection of IS tendon in the in vitro study. The gross observation also revealed that tendon ends of the transected tendon portion were distracted, which denoted that certain force applied to the lacerated IS tendon. However, this force did not exceed those required to rupture the remaining 2 mm intact tendon preserved. These result led us to speculate that this NWB canine model was a safe and reliable large animal model for a full-transection IS and repair for rotator cuff research.

Histologically, we noted abundant scar tissue filling the gap between the lacerated tendon ends, without any discontinuity. The scar also fused with the normal portion of the IS tendon, but the margin between normal tendon and scar could be clearly identified, even in gross observation. Tendon retraction and subsequent gap formation have been reported previously in animal models with full or partial weight-bearing after surgery[26,27], and in human patients, it is a major cause of failure after rotator cuff repair, with the tendon pulling through the suture[40,41].

In this study, we also compared the ultimate tensile strength of the modified Mason-Allen repair using different sutures. Either repair method provided greater strength than the 2-mm intact tendon without repair, but the construct repaired with the Fiberwire #2 suture had higher ultimate tensile load and stiffness than those repaired with the 3–0 Vicryl suture. These results illustrated that the suture material and size, in addition to suture techniques, are important factors that influence the mechanical strength of rotator cuff repair. However, our data also demonstrated that even with a relatively weaker repair using 3–0 suture materials, repair strength of the IS tendon exceeded the tensile force applied in the NWB canine model.

It should be recognized that the major limitation of this study was to use a partial laceration without repair to test the feasibility of this NWB model indirectly, instead of establishing a full laceration with repair in vivo model. Although the strength of the intact portion of the tendon in the partial laceration model was weaker than the full laceration repaired with #2 suture in vitro model, it should be cautioned there are differences in biomechanical and biological response between in vivo and in vitro model, and also between full and partial laceration models as well. This major limitation was because the dogs used in this study were primarily assigned for the other study that used NWB model, and the IACUC requested that the surgical procedure would be as simple as possible to minimize operative time and trauma. Therefore we designed this partial transection without repair with a small incision and quick procedure in vivo, and then using an in vitro model to study the mechanical strength of this partial transection compared to the repairs of a full transection model. However, the current study provided an indirect support for the feasibility of this novel NWB animal model for rotator cuff repair related research. We plan to move forward to verify the usefulness of the NWB model in a full laceration and repair model in the future. Second, partial weight-bearing and full weight-bearing models were not created, so we cannot compare the failure rate, ultimate tensile load, and stiffness of those models with the current NWB model. Third, a laceration was created at the tendon substance in this study, rather than one at tendon-to-bone insertion, which would introduce a clinically relevant scenario. However, the purpose of this study was to investigate if the strength of a 2-mm tendon bundle of the IS tendon could be tolerant a vivo force applied to the IS tendon without rupture, rather than the healing characteristics.

## Conclusion

We developed a canine NWB model for rotator cuff injuries by performing a radial neurectomy and using a loose sling. Our data showed the activities in the canine NWB model did not rupture a 2-mm intact portion of IS tendon, and the ultimate tensile load of this tendon portion was less than that of an IS tendon after full-width transection and repair with either 3–0 or #2 suture materials. Based on these data, we conclude that this canine NWB model for rotator cuff may be an appropriate and useful canine model for studies of rotator cuff repair.
